# The Relation of the Public Schools to the Medical Profession*Paper read by Arthur Lefevre, State Superintendent of Public Instruction, before the State Medical Association of Texas—Section on State Medicine and Public Hygiene, April 27, 1904.

**Published:** 1904-06

**Authors:** 


### TEXAS MEDICAL JOURNAL.

ESTABLISHED JULY, 1885.

PUBLISHED MONTHLY.—SUBSCRIPTION $1.00 A YEAR.

VOL. XIX. AUSTIN, JUNE, 1904. No. 12.

### Original Contributions.

For Texas Medical Journal.

### The Relation of the Public Schools to the Medical Profession.*

The growing spirit of mutual comprehension and co-operation between the various specialized functions of society is one of the most hopeful indications of progress toward the true aims of civilization. Every problem needs to be considered in the light of related problems, and only when workers in different spheres work with reciprocal sympathy and co-operation can they cease to work at cross purposes. Few men can successfully specialize in more than one department, but everywhere there is need to uphold the idea that specialization ought to be a matter of emphasis, not of exclusion. Relations of the public schools to the medical profession pointedly illustrate the universal principle to which I have alluded.

The health of the people is one of the prime responsibilities of statesmen; also, whatever needs to be developed throughout the life of a people needs to be introduced in the schools. These two propositions, separately acknowledged as they are, would have been more fruitful of practical results had each been dealt with in the light of the other, and both developed with intelligent recognition of their dependence upon medical science. Legislators, teachers and physicians must co-operate. It is not enough that they should be separately interested in the public health; in the

*Paper read by Arthur Lefevre, State Superintendent of Public Instruction, before the State Medical Association of Texas—Section on State Medicine and Public Hygiene, April 27, 1904.

2—Mj place of unco-ordinated and often mutually hindering efforts, should be substituted intelligent co-operation.

In the brief moments at my disposal, I can merely suggest a few practical measures by which far-reaching beneficial reforms might be inaugurated. Everything that I recommend has been tested, both as to feasibility and as to good effects, in the experience of various other States.

There are other matters, involving public health and protection against disease, where direct legislation is required; but for the immediate matter you have assigned for discussion to me, the only sort of legislation required is the provision of means to execute purposes already declared by statute, and to upbuild authority already conferred. For the most part, the spirit of the laws of Texas is statesmanlike, and in the main their general provisions challenge admiration; but the means provided for executing their purposes are too often absurdly inadequate. An institution "of the first class" is solemnly ordained; but appropriations commensurate with the purpose do not follow. A law wisely requires the public lands to be surveyed; but the surveyors could not perform the work in two centuries. The Constitution nobly commands that the Legislature shall supplement the income from the permanent school fund so that every public school may be maintained at least six months in each year; but even the one-fifth of 1 per cent tax permissible has never been fully levied, and the average school term for the common school districts which depend on State and county apportionments is less than five months. In like manner all needed authority is expressly conferred upon the State Superintendent of Public Instruction; but in some vital matters he is left legally powerless to fulfill duties imposed by law, and in others it is physically impossible with the appropriations made for the Department of Education.

In the matter of co-operating with a department of public health, all that the Department of Education needs, besides adequate appropriations for printing and distributing, is a law requiring the State Superintendent to withhold from any county or independent district amounts due from the State apportionment until reports, required according to law, have been duly received from local officers. The efficiency of the schools would not be thus endangered, because the withholding of a monthly payment would always insure immediate attention to neglected duties—duties imposed by existing laws in the most absolute terms, but left without means of enforcement. As a general proposition no public or private enterprise can afford to pay out great sums of money without exacting due reports both of the conditions and amounts of disbursements; and vital statistics are withheld which are essential to intelligent supervision. In particular, the mere power to enforce the making of suitable reports *would secure due attention to instructions* concerning, for instance, the examination of the eyesight of pupils, or of disinfecting city school buildings, issued in accordance with authority already amply conferred. A little money and the simple enactment suggested would place the Department of Education in a position to correct many existing unlawful and injurious conditions, and to discharge many useful functions otherwise impracticable.

On the side of the department of health, it seems to me that a bureau for the preparation of bulletins containing subject-matter suitable for distribution by the Department of Education, is the chief prerequisite for an effective dissemination through the public schools of the information needed for the self-protection and self-preservation of the people.

Infectious and communicable diseases are the causes of the premature death of thousands, and the still larger number who survive the primary diseases suffer permanently with their sequelæ. The greater part of this is preventable by means so simple that they can be understood by the pupils of our schools. Through the children parents would become interested and enlightened, and far-reaching results would be quickly secured. It is wonderful how easily children can be interested and instructed in such matters, and how much they will accomplish in practical ways when thus aroused. It is likely that the school children of San Antonio will soon be the means of nearly exterminating mosquitoes and flies in that city, thus saving themselves and their less docile fellow citizens from incalculable danger and suffering.

The school, especially the public school, is a focus to which all contagious diseases—diphtheria, scarlet fever, measles, whooping cough, sore throat, sore eyes, etc.—converge from infected homes, and from which they diverge outward to other homes. In our cities there should be, and easily could be, such daily medical inspection as is already practiced in some cities of Europe and of this country. Every morning the teachers could send to a room provided for the purpose such pupils as showed suspicious symptoms. There the medical inspector disposes of them according to his judgment. The medical inspectors of Boston, in fourteen months, thus examined 16,790 pupils, finding 10,737 cases of more or less serious sickness, of which 77 were diphtheria, 28 scarlatina, 116 measles, 28 chickenpox, 69 pediculosis, 47 mumps, 47

scabies, 33 whooping cough, over 2000 cases of follicular tonsilitis, and an equally number of contagious diseases of the eye. One examiner's report for one week in New York shows 364 children excluded, as follows: Measles 2, diphtheria 13, scarlet fever 1, croup 3, whooping cough 4, mumps 10, chickenpox 15, contagious diseases 59, skin diseases 19, parasitic diseases 227.

The extent of the evils thus prevented is incalculable, but the cost is very little. In Boston well qualified physicians serve at $200 a year, each inspector stopping at four buildings every morning on school days. The work on the part of the teachers is a simple matter consuming no appreciable time, and the visiting physician easily examines the children sent to the waiting room. If a child is found to be too ill from any cause to remain in school, the inspector advises the teacher to send him home to be cared for by parents and family physician, and if the sickness is caused by a contagious disease, he orders the child home and reports the case to the board of health, which promptly take steps for suitable isolation, should the case require such measures.

In villages and country schools the teachers could easily be taught enough of sanitary science and of the premonitory symptoms of many diseases, to accomplish much good and avert much evil, if the simple means already suggested of securing systematic attention to instructions were provided.

I may add that much good service might be rendered even without the preparation of bulletins by a department of public health. Of course, a competent bureau of such a department could prepare much more and better material, more timely to current conditions, and especially adapted to Texas; but with a small addition to office force and with a slight increase of appropriation the Department of Education would stand ready to begin the work.

I know noble men of science who would help with the generosity characteristic of their vocation; and for more than a year past only the lack of a few hundred dollars has prevented me from issuing instructions to all public school officers and teachers concerning various sanitary measures, and particularly requiring reports on the eyesight and hearing of pupils in accordance with simple means of examination, supplied with the instructions, that would enable any teacher to detect the existence of trouble and report the same to parents with the recommendation that medical advice be sought. I know that a law which merely declares a duty without providing any penalty for its neglect is for the most part *vox et preterea nihil;* nevertheless, had the pecuniary means been available, I would ere this have made such an attempt.

I believe that the next Legislature will not only make the small addition to the appropriation for printing and distributing needed, but that it will amend the statutes requiring teachers to make such reports to county superintendents as they may call for, and requiring the superintendents and treasurers of counties and independent districts to make such reports to the State Superintendent as he may call for,—by requiring county superintendents to withhold approval of vouchers until overdue reports are satisfactorily rendered, and by requiring the State Superintendent to withhold from any county or independent district amounts due from the State apportionment until reports required according to law have been received. There are a few treasurers of school funds in this State who make reports to nobody. The omission occurs not from defiance of the law, but from sheer negligence. The same thing occurs with statistical and other reports on the part of a few school superintendents, and the latter continually complain about the neglect of teachers. It ought to be obvious that efficient supervision according to duties and responsibilities imposed by law upon county superintendents and State Superintendent, are thus rendered impossible of fulfullment, and that many opportunities for service are thus destroyed.

Since co-operation is the theme of these remarks, may I not suggest that we help one another? If you, gentlemen of the Medical Association of Texas, will take the trouble to urge the men who *represent you* in the making of the laws, to give heed to the simple and unobjectionable remedial measures sought by the schools, then the schools in their turn will powerfully subserve many of your own great and noble endeavors. Gradually the idea you cherish will filter out from the schools into the homes, there to find practical application in every-day life. The people would then soon learn that successful sanitation requires the intelligent co-operation of all, and that effective protection against many plagues and dangers of life are under such conditions easily secured. On the one hand, teachers rate too low both their duties and their opportunities, and on the other hand the general public is prone to misconceive the schools as a burden for whose support they are taxed, instead of discerning that any proper taxation is simply a means of *co-operation* for some useful purpose. All discussion that tends to elevate the ideal or the tone, or to rectify the misconceptions of the other is serviceable.

But our people are already progressing rapidly in such matters —everything in the commonwealth of Texas is progressing rapidly—and it will not be so difficult as some think to prepare them.

to co-operate with every proper measure that contributes to the public welfare. The campaign of education should be prosecuted by our joint forces with hopeful energy. The spirit of the times strongly invites us.

[A case in point gives striking emphasis to the fourth paragraph of Professor Lefevre's splendid paper. The State Medical Association committee (of which the editor of the TEXAS MEDICAL JOURNAL and now President of the Association, was chairman, and State Health Officer Tabor was a member) secured the passage by the last Legislature of an act creating a Bureau of Vital Statistics in the Public Health Department. It provided for a registrar, or superintendent, and the small appropriation of $2000 a year to inaugurate the reform. On its final passage in the Senate the registrar and the little appropriation were cut out, mainly through the influence of that remarkable broad and enlightened statesman, Senator Travis Henderson, whose mediæval notions of "economy" would be ludicrous if not so disastrous, with the result that the law is inoperative and a dead letter.—EDITOR.]

